# Temporal, Spatial, and Environmental Variation in Virus-like Particle Abundance in the Northwestern Arabian Gulf

**DOI:** 10.3390/v18090969

**Published:** 2026-09-03

**Authors:** Awatef Almutairi, Dhia Al-Bader, Mashael Al-Mutairi

**Affiliations:** 1 Department of Biological Sciences, Faculty of Science, Kuwait University, P.O. Box 5969, Safat 13060, Kuwait; 2 Department of Medical Laboratory Sciences, Faculty of Allied Health Sciences, Kuwait University, P.O. Box 31470, Sulaibekhat 90805, Kuwait

**Keywords:** marine viruses, virus-like particles, viral abundance, cyanophage, *Synechococcus*, dissolved oxygen, nutrients, random forest, generalized additive model, Arabian Gulf

## Abstract

Marine viruses are important components of microbial communities and influence their structure and dynamics, yet their variability remains poorly documented in the northwestern Arabian Gulf. Virus-like particle (VLP) abundance was monitored over four years at three coastal sites representing different environmental settings in Kuwait. Surface and depth samples were collected during each sampling period, along with physicochemical and nutrient data. VLP abundance fluctuated throughout the study and differed among sites and between depths. VLP counts tended to be higher at the southern site and near the surface, while the lowest values were recorded in Kuwait Bay. The three sites had distinct environmental characteristics, but relationships between VLP abundance and individual physicochemical variables varied among sites and seasons. When environmental, spatial, and temporal variables were considered together, Random Forest identified season, dissolved oxygen, and nutrients, particularly nitrate, among the important predictors of VLP abundance. Dissolved oxygen showed a nonlinear association with VLP abundance in the generalized additive model (GAM), and lower abundance at depth remained significant after accounting for the measured environmental variables, while site effects were not significant in this model. Overall, VLP abundance showed marked spatial and temporal variability across Kuwait coastal waters, with patterns associated with multiple environmental and seasonal factors. This four-year record provides a baseline for viral abundance in the northwestern Arabian Gulf and supports further investigation of the biological and environmental processes influencing viral dynamics in this highly variable coastal system.

## 1. Introduction

Marine viruses are the most ubiquitous and diverse biological entities in the ocean and their abundance is typically one order of magnitude higher than that of bacteria [1,2], and viral lytic infections play a key role in shaping the composition and abundance of microbial communities [3,4]. Viruses, and cyanophages in particular, can influence marine primary productivity through infection of the abundant picocyanobacteria *Synechococcus* and *Prochlorococcus* [2,5]. Viruses influence oceanic biogeochemical cycles through the viral shunt, which returns host-derived organic matter to the microbial food web [2,6,7]. Viral particles may also be removed or degraded, releasing nutrient-rich material to the dissolved organic matter pool [8,9].

Marine viral abundance is usually estimated by enumerating virus-like particles (VLPs) using epifluorescence microscopy, transmission electron microscopy, or flow cytometry [10,11,12]. Although methods such as infectivity assays and molecular approaches targeting specific viral taxa provide information on viral infectivity, diversity, and the abundance of selected viral groups, they do not provide information on the total number of virus particles in environmental samples [13]. Consequently, VLP enumeration remains the standard method for estimating the standing stock of viruses in marine ecosystems [11,14].

Marine virus abundance varies spatially and temporally [15,16]. In short-term surveys, viral abundances vary with season, diel cycle, and depth [17,18,19], while longer time series report recurring seasonal and interannual patterns [20,21]. More recently, an 18-year time series from the northwestern Mediterranean distinguished recurring seasonal variability from persistent ecological change [22]. Environmental factors, including temperature, salinity, light, nutrient availability, and turbidity can influence these processes by altering host physiology, viral replication, virion stability, and particle retention [23,24]. However, the direction and strength of these effects vary among marine systems because environmental factors interact with local host communities, water-column structure and viral loss processes [17,20].

Recent metaviromic approaches have substantially expanded our understanding of marine viral communities by revealing viral diversity, temporal changes in community composition, previously uncultivated viral populations, and potential virus–host associations [25,26]. Metagenomic approaches can also provide sequence-based estimates of viral abundance and virus-to-microbe ratios, complementing particle-based measurements [27]. However, direct VLP enumeration remains valuable for quantifying total viral standing stocks independently of viral taxonomic assignment and for tracking changes in overall particle abundance. Combining direct enumeration with metaviromic approaches can therefore provide complementary information on viral abundance and community composition.

The northwestern Arabian Gulf is a shallow, semi-enclosed marine system in the northwestern Indian Ocean [28,29]. The region has an arid climate and persistently high salinity [30]. Evaporation greatly exceeds freshwater input across much of the Gulf, and exchange with the open ocean is restricted [28,31]. Together with shallow bathymetry, this restriction contributes to strong environmental gradients over short distances [28,30]. Kuwaiti coastal waters also receive freshwater and nutrient-rich inputs from the Shatt Al-Arab River [30,32]. Northern coastal waters contain fine sediments that are readily resuspended by winds and tidal currents, creating a north–south turbidity gradient, while water clarity generally increases toward the southern coast [30,33]. Such strong environmental gradients make the northwestern Arabian Gulf an ideal natural system for examining relationships between environmental variability and marine viral abundance.

Most viral work in the northwestern Gulf has focused on cyanophages. Analysis of the cyanophage g20 gene in Kuwaiti waters showed a diverse community that changed over time [34]. A later g20 amplicon study showed a phylogenetically diverse but highly uneven cyanophage community [29]. Despite these recent advances, the environmental drivers of total VLP abundance in the northwestern Arabian Gulf remain poorly characterized. Here, we used a four-year field dataset to examine seasonal, spatial, and vertical variation in VLP abundance across environmentally contrasting coastal waters of Kuwait. Multivariate and machine-learning approaches were used to identify environmental variables associated with these patterns and evaluate their relative importance.

## 2. Materials and Methods

### 2.1. Study Area and Sampling

**Figure 1:**
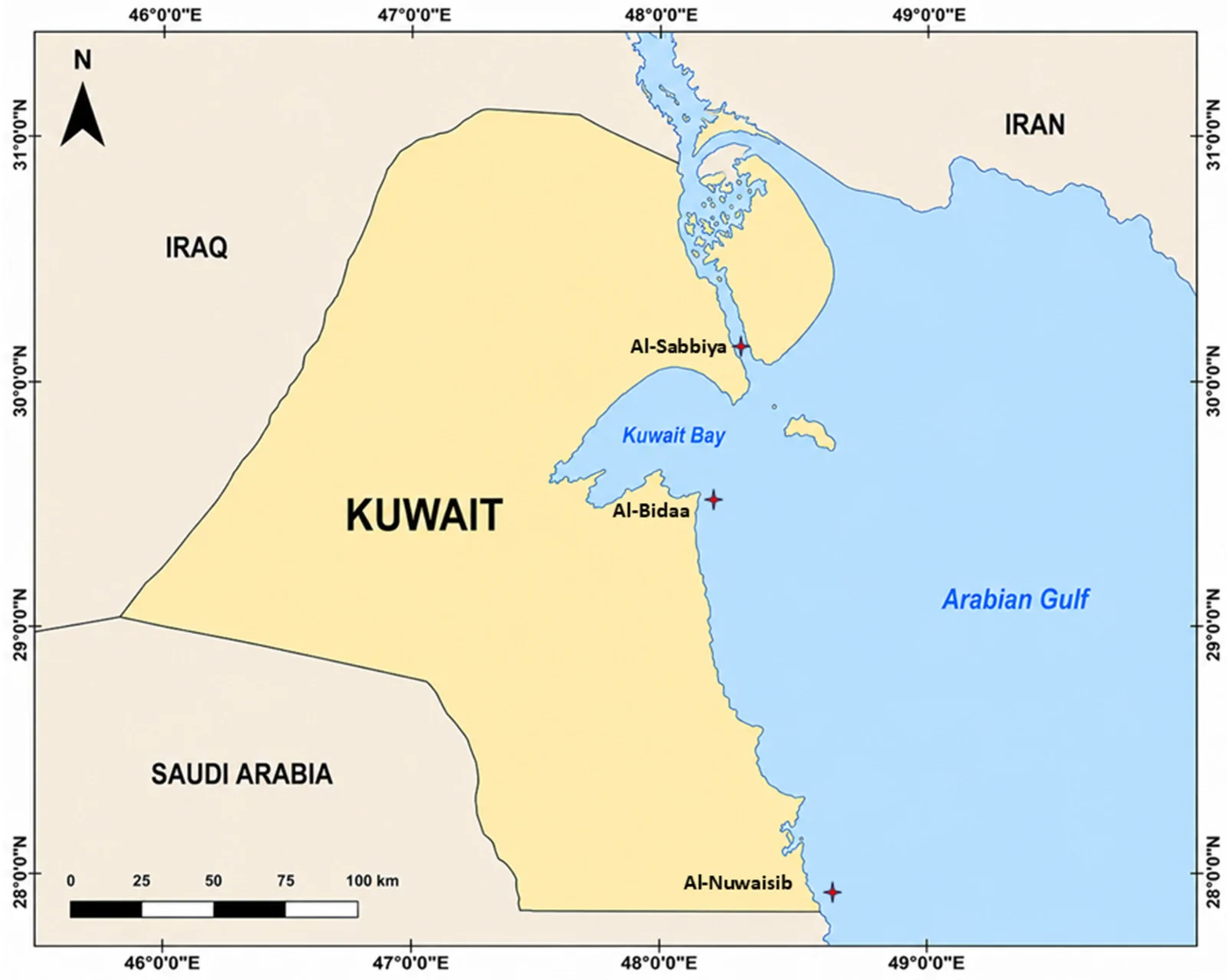
Location of the three coastal sampling sites along the Kuwaiti coast in the northwestern Arabian Gulf: Al-Sabbiya (SUB), Al-Bidaa (BID), and Al-Nuwaiseeb (NUW).

Seawater samples were collected from three coastal sites distributed along the Kuwait coast, representing a north-to-south environmental gradient (Figure 1). Al-Sabbiya (SUB; 29.580689° N, 48.170426° E) is located in northern Kuwait and is influenced by freshwater discharge and suspended sediments from the Shatt Al-Arab system. Al-Bidaa (BID; 29.325947° N, 48.08437° E) is located along the central Kuwait coast in an area influenced by urban coastal activities. Al-Nuwaiseeb (NUW; 28.547482° N, 48.432662° E) is located in southern Kuwait and is characterized by relatively clear, more oceanic waters.

Sampling was conducted bimonthly from April 2011 to January 2014 at the surface and at 2 m depth at each site, following the sampling strategy described previously [34]. Seawater samples were collected concurrently for physicochemical measurements, nutrient analysis, and virus-like particle (VLP) enumeration. For VLP enumeration, approximately 50 mL aliquots of seawater were collected and fixed immediately with 0.02 µm-filtered formalin (37–39% formaldehyde solution) to a final concentration of 2% (*v*/*v*), following Patel et al. [12]. The fixed samples were flash-frozen in liquid nitrogen and stored at −80 °C until analysis.

### 2.2. Physicochemical Parameters and Nutrient Analysis

At each sampling site, seawater temperature, salinity, pH, dissolved oxygen (DO), and turbidity were measured using a multiparameter water-quality meter (Horiba Ltd., Kyoto, Japan). Seawater samples were collected concurrently for dissolved inorganic nutrient analysis. Nitrate (NO_3_^−^), nitrite (NO_2_^−^), ammonium (NH_4_^+^), phosphate (PO_4_^3−^), and silicate (SiO_3_^2−^) were quantified spectrophotometrically using a Cary 5 UV–Vis–NIR spectrophotometer (Varian Australia Pty Ltd., Mulgrave, VIC, Australia). Nitrate, nitrite, and phosphate were analyzed using NANOCOLOR VIS methods, whereas silicate was determined using the silicomolybdo-complex method at 810 nm and ammonium using the indophenol method at 630 nm. Nutrient concentrations were expressed as µmol L^−1^.

### 2.3. Enumeration of Virus-like Particles

Virus-like particles (VLPs) were enumerated by epifluorescence microscopy following Patel et al. [12]. Preserved samples were thawed immediately before analysis and thoroughly mixed before subsampling. A 250 µL aliquot was diluted to 1 mL with 0.02 µm-filtered seawater and filtered through a 0.02 µm Anodisc membrane filter (Whatman, Maidstone, UK). Sample preparation and filtration were performed in triplicate. Filters were stained with diluted SYBR Green I (Life Technologies, Eugene, OR, USA) for 15 min in the dark. Excess stain was removed, and the filters were mounted on glass microscope slides using 0.1% (*v*/*v*) p-phenylenediamine antifade solution.

VLPs were enumerated using a Zeiss Axioskop epifluorescence microscope (Carl Zeiss, Oberkochen, Germany) equipped with a 100× oil-immersion objective and a blue excitation filter set (450–490 nm). VLPs were identified as small, discrete fluorescent signals, whereas clearly larger fluorescent signals corresponding to bacterial cells or particulate and aggregated material were excluded from the counts. At least 20 randomly selected fields were examined per filter, with a minimum of 200 VLPs counted. VLP abundance was calculated from the triplicate counts and expressed as VLPs mL^−1^.

### 2.4. Statistical Analysis

All statistical analyses and graphical visualizations were performed in R version 4.5.1 [35]. Unless otherwise stated, statistical significance was assessed at α = 0.05. Principal component analysis (PCA) was performed on standardized environmental variables (temperature, salinity, turbidity, pH, and dissolved oxygen). Variables were centered and scaled to unit variance, and PCA was conducted using the prcomp function in the stats package (version 4.5.1). Environmental variables were fitted onto the ordination using the envfit function in the vegan package (version 2.7-5) [36], with significance assessed using 999 permutations.

Relationships between VLP abundance and environmental variables were examined using Spearman’s rank correlation. Correlations were calculated separately by sampling site (SUB, BID, and NUW), water-column position (surface and depth), and season for temperature, salinity, turbidity, pH, dissolved oxygen, and ammonium. Seasonal correlations were based on three averaged observations for each site–season–depth combination and were therefore treated as exploratory indicators of association. Correlation coefficients and associated *p*-values were calculated using the Hmisc package (version 5.2-6), with data processing performed using dplyr (version 1.2.1) and tidyr (version 1.3.2). To account for multiple comparisons, *p*-values were adjusted using the Benjamini–Hochberg false discovery rate (BH–FDR) procedure [37]. Adjusted q-values < 0.05 were considered statistically significant. Correlation coefficients and BH–FDR-adjusted significance levels were visualized as heatmaps using the pheatmap package (version 1.0.13).

Differences in log_10_-transformed VLP abundance among sampling sites were assessed using the Kruskal–Wallis test. Significant differences were further examined using Dunn’s post hoc test with Bonferroni correction, and effect size was expressed as epsilon-squared (ε^2^). Differences between surface and depth samples were evaluated using the Wilcoxon rank-sum test, with effect size reported as r. These analyses were performed using the FSA (version 0.10.1) and rstatix (version 0.7.2) packages.

Random forest regression was used to evaluate the relative importance of environmental, nutrient, spatial, and temporal predictors of log_10_-transformed VLP abundance. The nutrient-inclusive model included temperature, salinity, turbidity, pH, dissolved oxygen, NH_4_^+^, NO_2_^−^, NO_3_^−^, PO_4_^3−^, SiO_3_^2−^, sampling site, water-column position, year, and season. The analysis was restricted to complete observations (*n* = 101). Models were fitted using the randomForest package (version 4.7-1.2) [38] with 2000 trees and four variables randomly selected at each split. Predictor importance was assessed using permutation importance (%IncMSE) and increase in node purity (IncNodePurity), while predictive performance was evaluated using the coefficient of determination (R^2^) and root mean square error (RMSE). Collinearity among nutrient predictors was examined using Spearman rank correlations on the same complete-case dataset (*n* = 101), with *p*-values adjusted for multiple comparisons using the Benjamini–Hochberg false discovery rate (FDR) procedure. A second, nutrient-excluded model containing the environmental, spatial, and temporal predictors was fitted to assess model performance in the absence of nutrient variables. Performance of the two models was compared using R^2^ and RMSE.

Generalized additive models (GAMs) were fitted using the mgcv package (version 1.9-4) [39] to examine nonlinear relationships between log_10_-transformed VLP abundance and environmental conditions. Temperature, salinity, turbidity, pH, and dissolved oxygen were included as smooth terms using thin-plate regression splines (k = 4), while sampling site and water-column position were included as categorical predictors. Candidate models incorporating alternative spatial and temporal structures, including site, water-column position, season, year, and a cyclic smoother for sampling month, were initially fitted using maximum likelihood (ML) and compared using Akaike Information Criterion (AIC). Models with ΔAIC < 2 were considered to have comparable support; when multiple models met this criterion, the model with the lowest effective degrees of freedom (EDF) was selected as the more parsimonious model. The selected model was subsequently refitted using restricted maximum likelihood (REML) for parameter estimation and statistical inference.

GAM performance was evaluated using adjusted R^2^, deviance explained, and RMSE. Model assumptions were assessed using quantile–quantile, residual-versus-fitted, and residual distribution plots. The adequacy of the smooth basis dimensions was evaluated using gam.check(), and predictor concurvity was assessed using concurvity(). Partial-effect plots were generated using the gratia package (version 0.11.2). To assess whether the inclusion of Season affected inference for the environmental smooth terms, a sensitivity analysis was performed by refitting the selected model without Season while retaining the same environmental smooth terms, site, and water-column position. Concurvity and the significance of the environmental smooth terms were then compared with those of the selected model.

### 2.5. Illustrative Experimental Evaluation of Suspended-Sediment Effects on Cyanophage–Host Interactions

To provide an illustrative laboratory assessment of the potential influence of suspended sediment on cyanophage–host interactions, an infection experiment was conducted using cyanophage KLS-01 and *Synechococcus* sp. WH7803 as the host. Cyanophage KLS-01 was originally isolated from Kuwait coastal waters using *Synechococcus* sp. WH7803. Locally isolated cyanobacterial strains were not used because axenic cultures were not available for the controlled infection experiment. Exponentially growing *Synechococcus* cultures were maintained in ASN medium at 25 °C under a 13:11 h light–dark cycle with continuous shaking. The initial host–cell density was approximately 4.6 × 10^6^ cells mL^−1^ and was measured before the addition of cyanophage or sediment (Day 0).

Sediment collected from the Al-Sabbiya coastal area, Kuwait, was dried and added directly to the ASN medium, following the general approach of Barnet et al. [40], with modifications. Sediment additions were adjusted to produce turbidity levels based on the mean value reported for Kuwait coastal waters (32.6 NTU) [30]. Three treatments were established: 32.6 NTU (1×), 48.9 NTU (1.5×), and 65.2 NTU (2×). Purified cyanophage KLS-01 (1.8 × 10^8^ particles mL^−1^ in the phage preparation) was added to each sediment treatment and to a positive control without added sediment. *Synechococcus* cultures without cyanophage or added sediment served as the negative control. All treatments and controls were performed in triplicate and maintained under identical incubation conditions. Host–cell abundance was monitored daily using a haemocytometer and epifluorescence microscopy (Zeiss, Germany) following Guillard and Sieracki [41].

## 3. Results

### 3.1. Temporal and Seasonal Variation in Virus-like Particle Abundance

**Figure 2:**
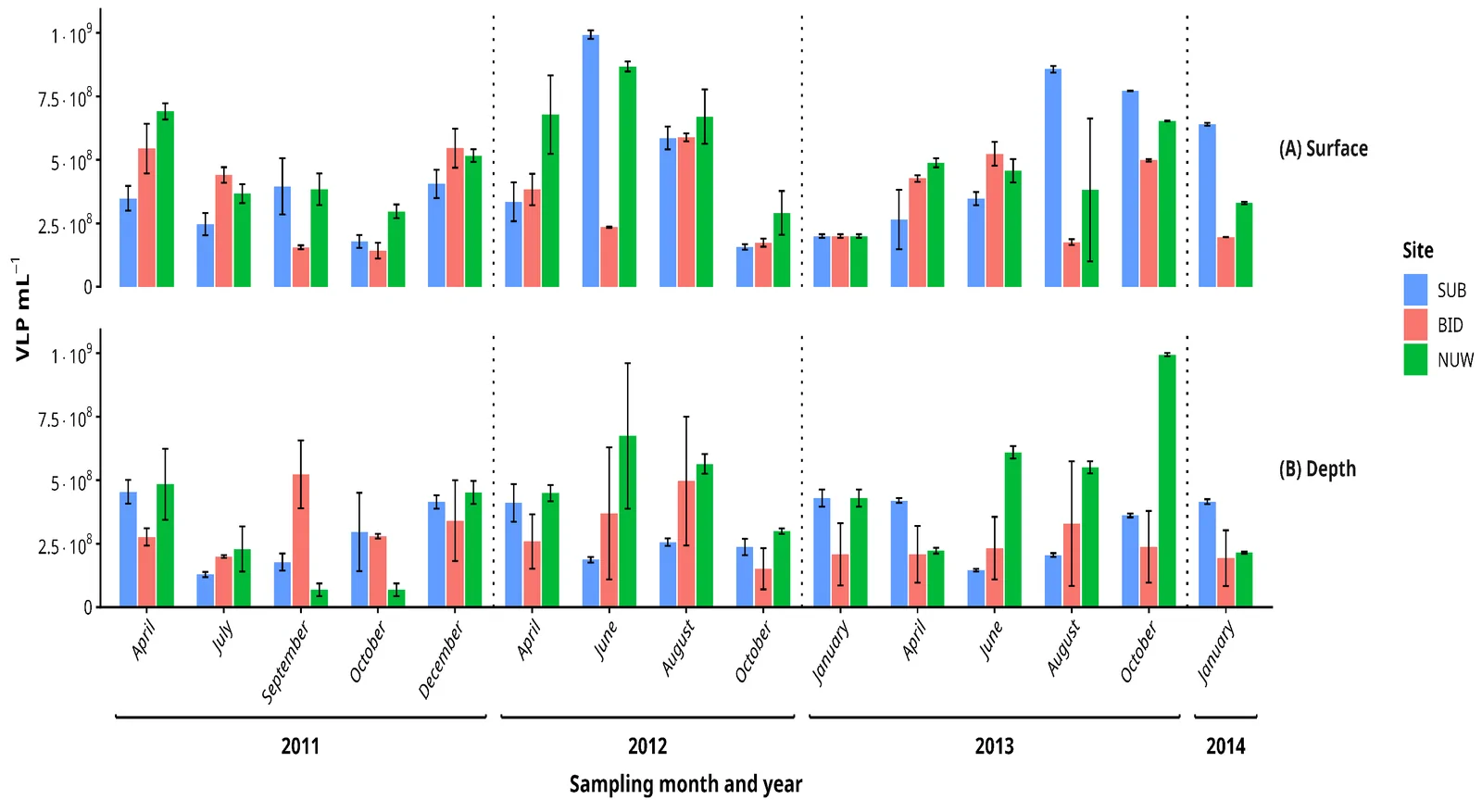
Temporal variation in virus-like particle (VLP) abundance at the three sampling sites from 2011 to 2014. VLP abundance (VLP mL^−1^) is shown for (**A**) surface and (**B**) depth samples at SUB, BID, and NUW. Bars represent mean values, and error bars indicate standard error (SE). Vertical dotted lines separate sampling years.

Virus-like particle (VLP) abundance changed throughout the study period (2011–2014) at all three sampling sites in both surface and depth waters (Figure 2A,B). Overall, SUB and NUW exhibited higher VLP abundances than BID throughout the study. Surface waters generally exhibited higher VLP abundances than depth waters, although this pattern varied seasonally among the three sites. Mean VLP abundance ranged from approximately 1 × 10^8^ to 1 × 10^9^ particles mL^−1^.

At SUB, surface VLP abundance was highest during the summer months (June–August) of 2012 and 2013, reaching a maximum of 9.93 × 10^8^ particles mL^−1^ in June 2012. During the colder months of 2012 and 2013, depth VLP abundance was generally higher than surface abundance. At BID, surface VLP abundance reached a maximum of 5.89 × 10^8^ particles mL^−1^ during summer 2012, whereas the highest depth abundance (5.23 × 10^8^ particles mL^−1^) was recorded in September 2011. At NUW, surface VLP abundance was high during 2011 and the first half of 2012, reaching 8.68 × 10^8^ particles mL^−1^ in June 2012. During 2013, VLP abundance at depth was generally higher than surface water abundance and reached the highest value recorded in the study (9.94 × 10^8^ particles mL^−1^) in October 2013, whereas surface abundance remained higher only in April 2013.

### 3.2. Environmental and Spatial Patterns Associated with VLP Abundance

**Figure 3:**
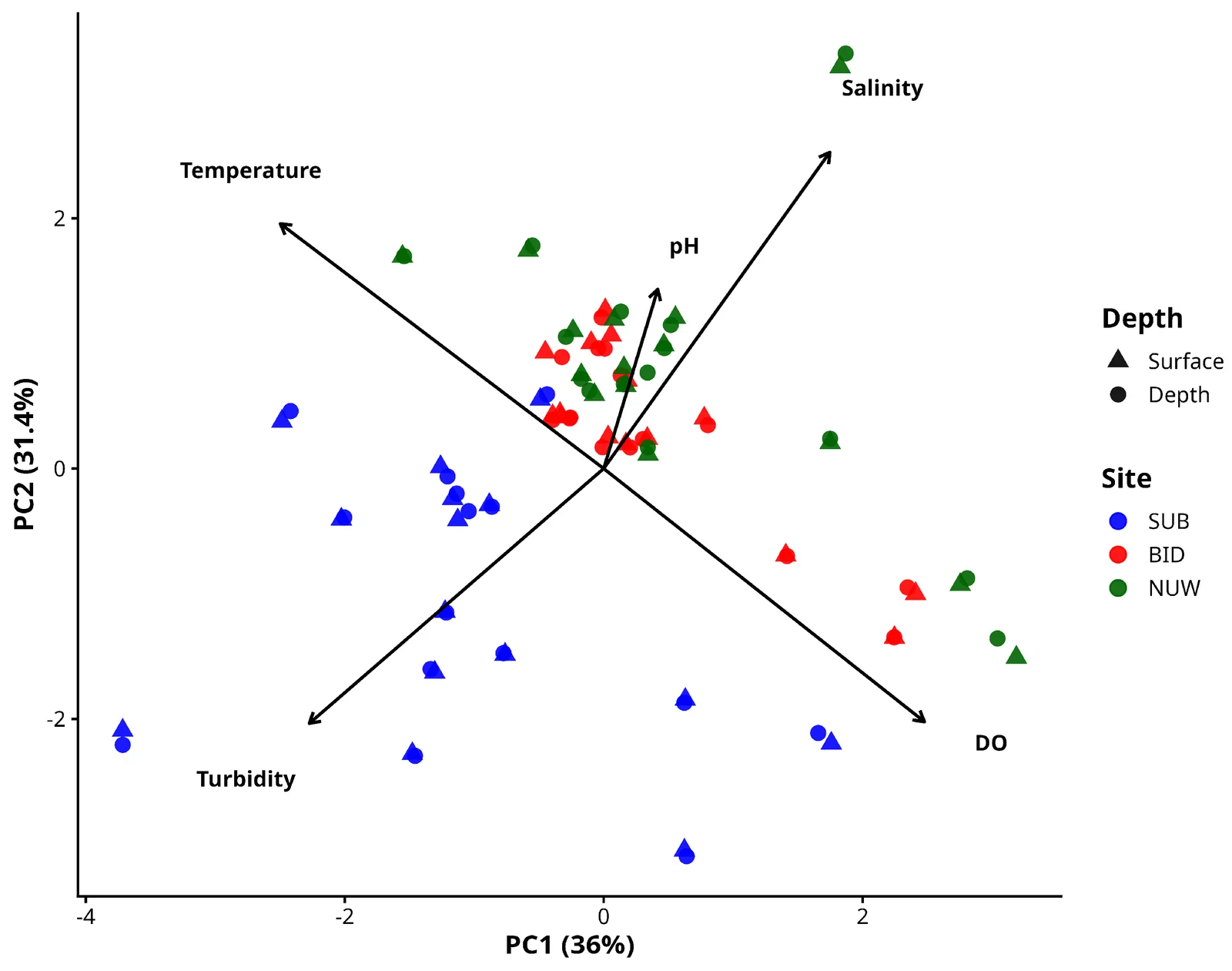
Principal component analysis (PCA) of environmental variables across the three sampling sites. Samples are colored by site and shaped by water-column position. Black arrows represent significant environmental vectors identified using envfit (999 permutations; *p* < 0.05).

Principal component analysis (PCA) summarized the environmental variation across sampling sites, seasons, and water-column positions (Figure 3). The first two principal components explained 67.4% of the total variation, with PC1 and PC2 accounting for 36.0% and 31.4%, respectively. Environmental fitting showed that dissolved oxygen, temperature, salinity, and turbidity were more strongly associated with the ordination, whereas pH showed a weaker but significant association (Table S1).

Although samples overlapped considerably, clear differences among sampling sites were evident. SUB samples clustered predominantly on the negative side of PC1, whereas NUW samples occurred more frequently on the positive side of the ordination. BID samples occupied intermediate positions between the two sites. Surface and depth samples overlapped extensively, indicating that sampling site contributed more to the observed environmental variation than water-column position.

Exploratory Spearman rank correlation analysis identified only six significant correlations between log_10_-transformed VLP abundance and environmental variables after Benjamini–Hochberg false discovery rate correction (Figure S1; Table S2). At BID, VLP abundance was negatively correlated with turbidity (ρ = −0.82) and temperature (ρ = −0.70) during summer at the surface, whereas ammonium showed a perfect negative correlation during winter at the same position (ρ = −1.00). At NUW, temperature was negatively correlated with VLP abundance during autumn at depth (ρ = −0.95). During summer at depth, dissolved oxygen was positively correlated with VLP abundance (ρ = 0.75), whereas ammonium showed a perfect negative correlation (ρ = −1.00). No significant correlations were detected at SUB after multiple-testing correction.

**Figure 4:**
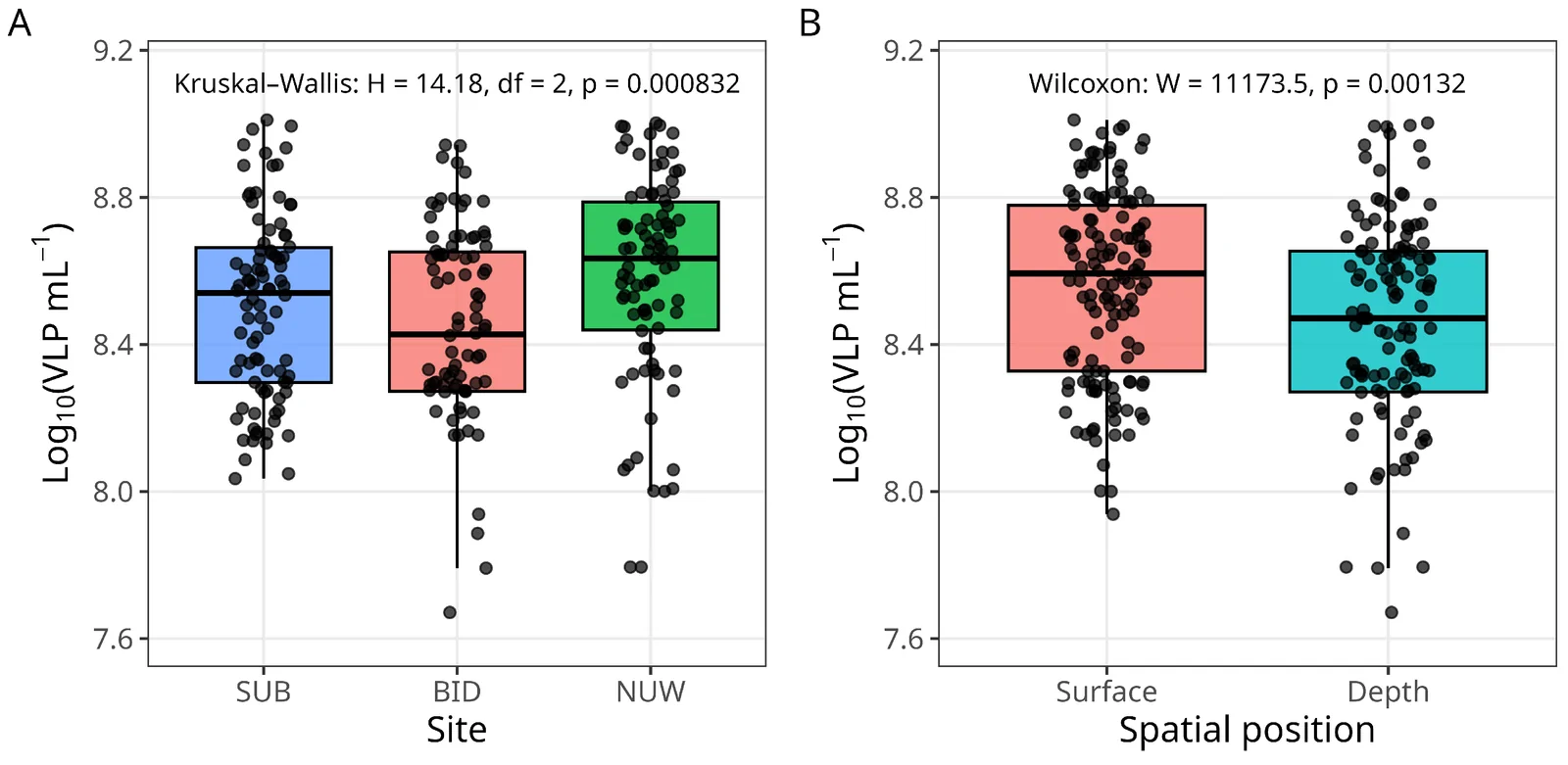
Spatial variation in log_10_-transformed virus-like particle (VLP) abundance. (**A**) Differences among sampling sites were assessed using the Kruskal–Wallis test. (**B**) Differences between surface and depth waters were assessed using the Wilcoxon rank-sum test. Points represent individual observations. Boxes indicate the median and interquartile range (IQR), horizontal lines indicate the median, and whiskers extend to 1.5 × IQR.

VLP abundance differed among the three sampling sites (Figure 4A). Median log_10_(VLP) abundance was highest at NUW (8.63), followed by SUB (8.54) and BID (8.43) (Table S3). The Kruskal–Wallis test detected significant differences among sites (H = 14.18, df = 2, *p* < 0.001; ε^2^ = 0.046). Dunn’s post hoc test showed that NUW had significantly higher VLP abundance than BID (adjusted *p* < 0.001), whereas differences between SUB and BID and between SUB and NUW were not significant (adjusted *p* > 0.05) (Table S4). VLP abundance also differed between water-column positions (Figure 4B). Surface waters had a higher median log_10_(VLP) abundance (8.59) than depth waters (8.47) (Table S3). The Wilcoxon rank-sum test confirmed this difference (W = 11,173.5, *p* = 0.0013; r = 0.196).

### 3.3. Random Forest Analysis of VLP Abundance Predictors

**Figure 5:**
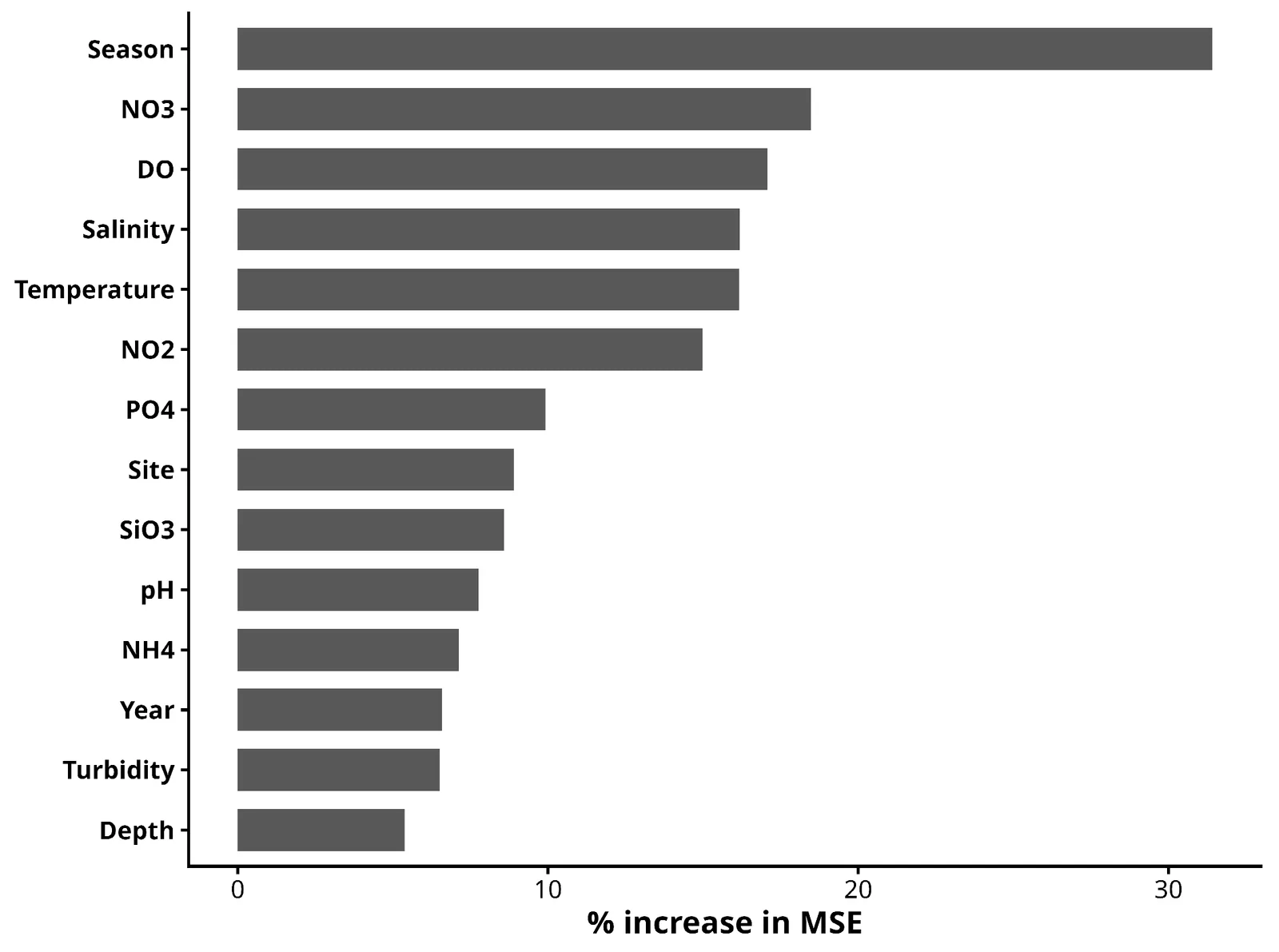
Random Forest permutation variable importance (% increase in mean squared error; %IncMSE) for the full model predicting log_10_-transformed virus-like particle (VLP) abundance. The model included environmental, nutrient, spatial, and temporal predictors.

A Random Forest model incorporating environmental, nutrient, spatial, and temporal predictors was fitted to the complete-case dataset (*n* = 101). The model explained 24.3% of the variation in log_10_-transformed VLP abundance (R^2^ = 0.243, RMSE = 0.312; Figure S2). Permutation importance (%IncMSE) ranked season highest, followed by nitrate and dissolved oxygen, with the remaining nutrient and environmental variables showing progressively lower importance (Figure 5). In contrast, variable importance based on increase in node purity ranked ammonium highest, followed by season and the remaining nutrient and environmental variables (Figure S3). Spearman correlation analysis of the nutrient predictors identified significant positive correlations between NH_4_^+^ and PO_4_^3−^ (ρ = 0.588), NH_4_^+^ and NO_2_^−^ (ρ = 0.494), and NO_3_^−^ and PO_4_^3−^ (ρ = 0.446), as well as a weaker negative correlation between NH_4_^+^ and SiO_3_^2−^ (ρ = −0.249); all remained significant after FDR correction (Table S5).

To assess the contribution of nutrient variables, a reduced Random Forest model excluding nutrient predictors was fitted while retaining the environmental, spatial, and temporal variables. In the absence of nutrient information, permutation importance ranked water-column position highest, followed by turbidity, site, and dissolved oxygen (Figure S4), whereas dissolved oxygen showed the highest node-purity importance (Figure S5). The reduced model showed substantially lower predictive performance than the nutrient-inclusive model (R^2^ = 0.100, RMSE = 0.411; Figure S6), indicating that inclusion of nutrient variables markedly improved prediction of VLP abundance.

### 3.4. Generalized Additive Model Analysis

Generalized additive models (GAMs) were compared using maximum likelihood (ML) to examine environmental, spatial, and temporal variation in VLP abundance (Table S6). The model containing environmental variables, site, water-column position, and season (M3) had the lowest AIC (148.61). The model additionally incorporating year (M5; ΔAIC = 1.44) and the alternative model using cyclic month instead of season (M6; ΔAIC = 1.67) also received substantial support, although M3 had the lowest AIC and was retained as the final model. M3 was selected and refitted using restricted maximum likelihood (REML) for parameter estimation and statistical inference.

**Figure 6:**
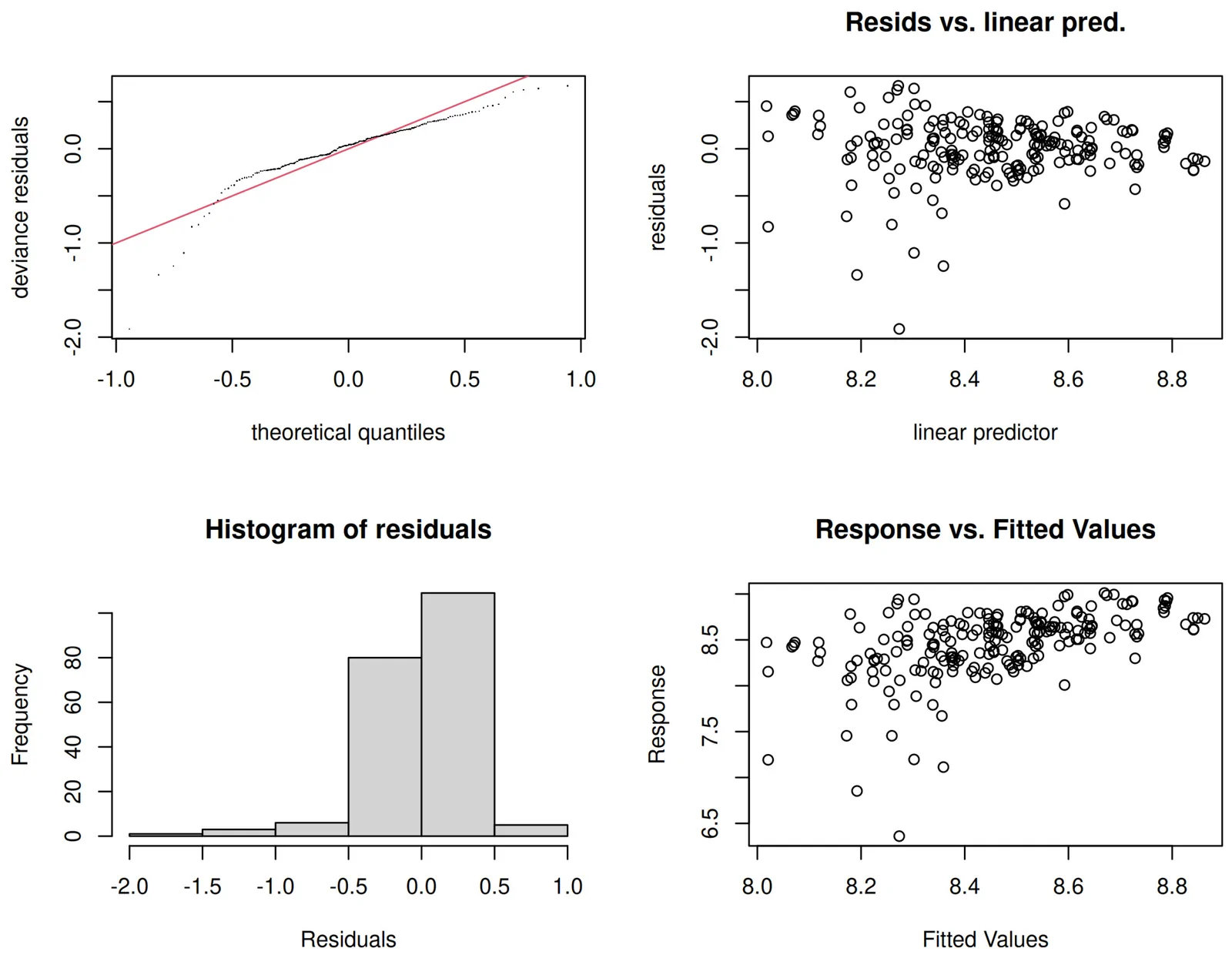
Diagnostic plots for the final generalized additive model (GAM). Panels show the quantile–quantile (Q–Q) plot of deviance residuals, with black points representing the residual quantiles and the red line representing the reference line (**upper left**), deviance residuals versus the linear predictor (**upper right**), histogram of residuals (**lower left**), and observed versus fitted log_10_-transformed virus-like particle (VLP) abundance (**lower right**).

**Figure 7:**
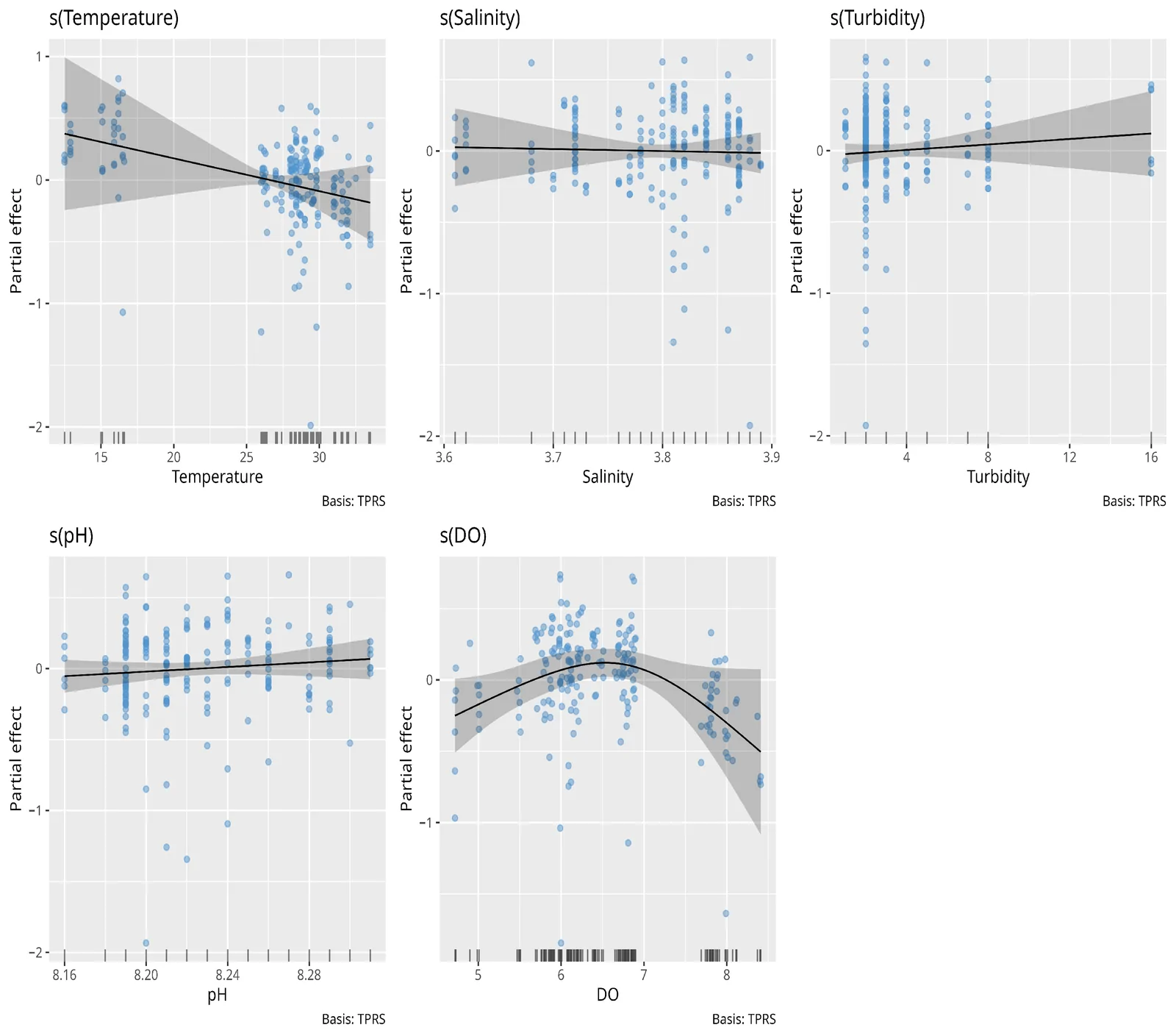
Partial-effect plots showing the estimated smooth relationships between environmental variables and log_10_-transformed virus-like particle (VLP) abundance in the final generalized additive model (GAM). Solid lines represent fitted smooth functions, shaded areas indicate 95% confidence intervals, and rug marks along the x-axes show the distribution of observations. The *y*-axis represents the partial contribution of each predictor after accounting for the remaining variables in the model. Statistical significance of the smooth terms is summarized in Table S9.

The final model explained 25.03% of the deviance in log_10_-transformed VLP abundance (adjusted R^2^ = 0.201; RMSE = 0.324; Table S7, Figure 6). VLP abundance was significantly lower at depth than at the surface (estimate = −0.201 ± 0.047, *p* < 0.001), whereas site effects were not significant (Table S8). Among the environmental smooth terms, dissolved oxygen was the only variable showing a significant nonlinear relationship with VLP abundance (EDF = 2.463, *F* = 3.184, *p* = 0.017; Figure 7; Table S9).

Model diagnostics were generally acceptable, although a small number of observations showed relatively large negative residuals. Concurvity was highest for temperature (estimate = 0.981), followed by dissolved oxygen (0.854) and salinity (0.836), and was lower for turbidity (0.594) and pH (0.579; Table S10). Because temperature and salinity vary seasonally in Kuwait waters, the model was also refitted without Season as a sensitivity analysis. Concurvity estimates were reduced to 0.932 for temperature, 0.824 for salinity, 0.548 for turbidity, 0.483 for pH, and 0.820 for dissolved oxygen. Dissolved oxygen remained the only significant environmental smooth term (*p* = 0.015). Thus, excluding Season generally reduced concurvity while leaving the significance pattern of the environmental smooth terms unchanged.

### 3.5. Effects of Suspended Sediment on Cyanophage–Host Interactions

**Figure 8:**
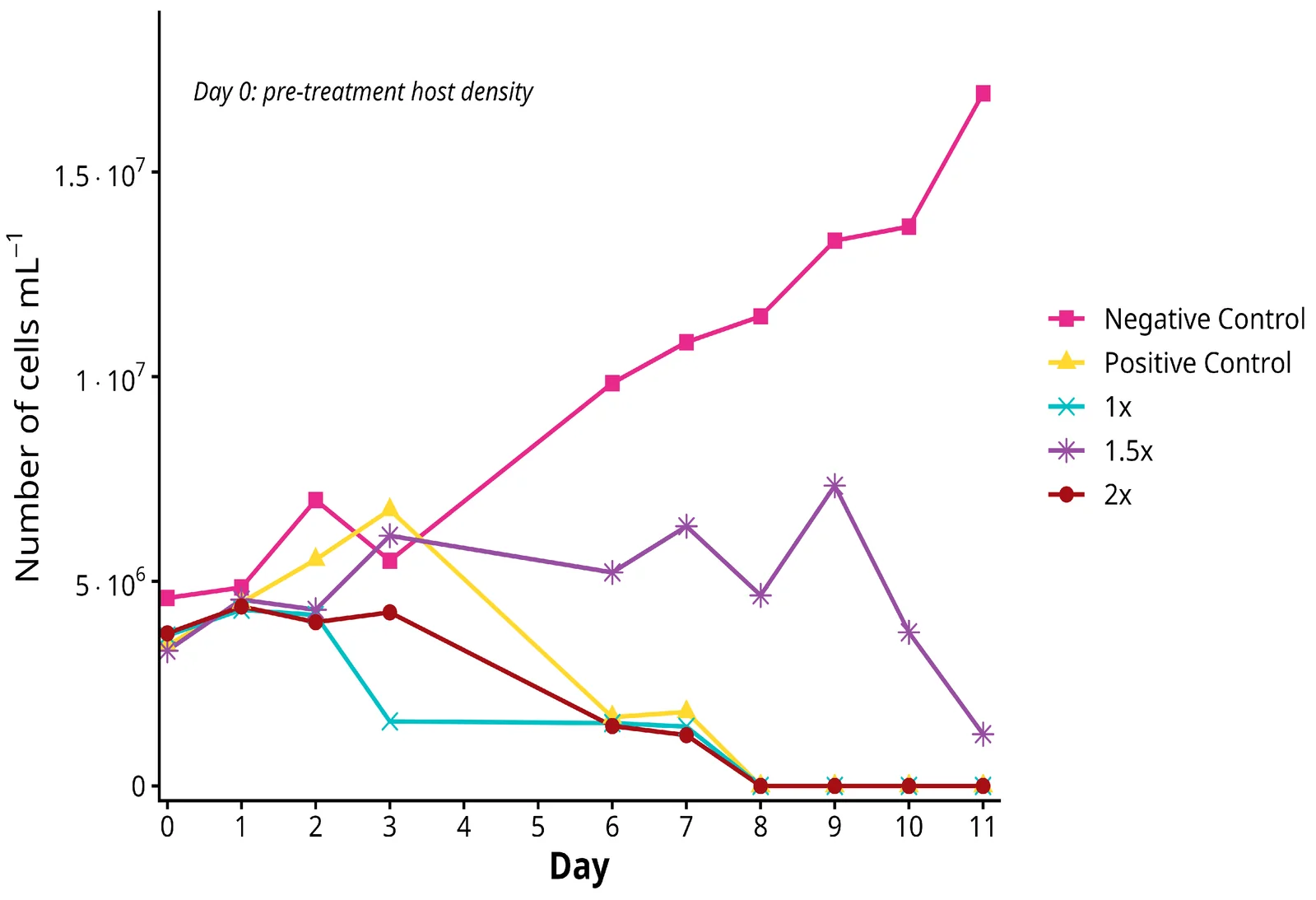
Effect of suspended sediment concentration on cyanophage-mediated lysis of *Synechococcus* sp. WH7803. Day 0 represents the initial host–cell density measured before the addition of cyanophage or suspended sediment. Cultures were subsequently infected with cyanophage KLS-01 and incubated without added sediment (positive control) or with suspended sediment at 1× (32.6 NTU), 1.5× (48.9 NTU), and 2× (65.2 NTU). The negative control consisted of uninfected cultures without cyanophage or added sediment. Host–cell abundance was monitored for 12 days.

To further examine the potential influence of turbidity on cyanophage–host interactions, an illustrative laboratory experiment was conducted using *Synechococcus* sp. WH7803 infected with the locally isolated cyanophage KLS-01 (Figure 8).

In the negative control, host–cell abundance generally increased throughout the experiment, reaching approximately 1.7 × 10^7^ cells mL^−1^ on the final sampling day. The positive control initially increased to 6.74 × 10^6^ cells mL^−1^ by day 4 and subsequently declined to zero by day 9. Similar declines occurred in the 1× and 2× sediment treatments, with host–cell abundance also reaching zero by day 9. In contrast, host–cell abundance remained higher in the 1.5× treatment during the later stages of the experiment, reaching 7.34 × 10^6^ cells mL^−1^ on day 10 before declining to 1.26 × 10^6^ cells mL^−1^ by day 12.

## 4. Discussion

VLP abundance in Kuwaiti coastal waters was similar to values reported from other productive coastal environments [24,42], with occasional higher peaks. Abundance varied by site, season, and water-column position and was generally higher at NUW and SUB, particularly during 2012–2013. PCA separated the sites according to differences in dissolved oxygen, temperature, salinity, and turbidity but showed considerable overlap between surface and depth samples.

The spatial distribution of VLPs did not correspond to the turbidity gradient. VLP abundance was lowest at BID, while the highest turbidity influence was reported farther north around Al-Sabbiya, and water clarity generally increases southward [30,33]. The low VLP abundance at BID may therefore reflect other conditions within Kuwait Bay. Limited water exchange and inputs of nutrients, sewage, industrial discharges, and other coastal sources have long affected the bay [32,43]. Ismail and Almutairi (2022) [44] also reported lower bacterial diversity and evenness in Kuwait Bay than in more open coastal areas. These environmental and microbial characteristics may contribute to the lower VLP abundance at BID. Correlations between VLP abundance and environmental variables differed among sites, seasons, and depths, further indicating that the spatial pattern was not associated with a single measured variable. Surface VLPs were usually more abundant than at deeper levels, even though principal component analysis (PCA) showed that surface and depth samples overlapped. This does not conflict with the greater cyanophage diversity previously reported at depth in Kuwait waters [34], because that study examined cyanophage diversity, while the present study measured total VLP abundance.

Random Forest and GAM were used to examine environmental, spatial, and temporal variation in VLP abundance. In the Random Forest model, nitrate, season, and dissolved oxygen were among the main predictors. Nitrate varied considerably during the study (19.17–2800 µg L^−1^). High and spatially variable nitrate concentrations have previously been reported in Kuwait waters, particularly in northern waters near Al-Sabbiyah, where our SUB site was located [45]. The high Random Forest ranking of nitrate should therefore be interpreted as predictive importance rather than evidence of a direct effect on VLP abundance. Nutrient availability can nevertheless influence viral abundance and community composition indirectly through effects on microbial host growth and activity [5,24]. The two Random Forest importance measures differed in the relative ranking of predictors, as expected because they quantify importance differently and can be affected by correlations among predictors. Nitrate ranked higher by permutation importance, whereas ammonium ranked highest by node-purity importance. Permutation importance was used as the main indicator of predictor relevance because it measures the loss of model performance when each variable is permuted.

In the GAM, dissolved oxygen was the only measured environmental variable significantly related to VLP abundance, with a nonlinear response. DO reflects several biological and physical processes that can influence microbial hosts and, in turn, viral production [15]. In Kuwait waters, *Synechococcus* abundance varies seasonally and has been positively associated with DO [44]. Changes in oxygen conditions have also been associated with shifts in microbial and viral communities in other marine environments [46,47]. Temperature and salinity also vary seasonally in Kuwait waters [30,44], but neither was significant in the GAM. Removing Season from the model did not change this result, indicating that the DO relationship was not simply a consequence of the seasonal term. Similar patterns have been reported in long-term coastal studies, where viral abundance varied with season and with several environmental and biological factors [22]. VLP abundance in our study also remained significantly lower at depth after accounting for the measured environmental variables. Because host-specific viral production was not measured and oxygen concentrations were not hypoxic, these data cannot resolve the processes behind the DO relationship or the surface–depth difference.

The sediment experiment showed that suspended particles affected cyanophage–host interactions. Particle attachment can reduce contact between viruses and susceptible hosts [48], while reduced cyanophage-mediated lysis and delayed viral infection following adsorption to sediment have also been reported [40,49]. Turbidity in the experimental treatments exceeded the average values reported for Kuwait coastal waters, although similar levels have been recorded locally [30,50]. The response did not increase with sediment concentration. Host–cell abundance was higher in the 1.5× treatment (48.9 NTU) than in the other treatments during the later stages. This may reflect temporary adsorption of cyanophages to particles followed by their release into the water [49], although free and particle-associated viruses were not measured separately. Thus, the experiment shows that suspended sediment can affect cyanophage–host interactions under turbidity conditions relevant to Kuwait waters, but it does not establish the mechanism or explain the field relationship between turbidity and total VLP abundance.

Several limitations should be considered. Epifluorescence microscopy (EFM) estimates total VLP abundance but cannot identify viruses or their hosts or determine infectivity [12,51,52]. Counts may also be affected by staining efficiency, background fluorescence, particle aggregation, suspended material, and manual identification of fluorescent particles [52,53,54]. Although SYBR Green I stains particles that contain nucleic acid and has low affinity for sediment particles, high particulate loads may obscure fluorescent particles or promote aggregation [12]. To reduce variability, we thoroughly mixed the samples before taking subsamples, diluted them before filtration, and counted in triplicate using a standardized procedure. Suspended particles may have contributed to the lower VLP counts at depth, but we did not independently quantify particulate matter and therefore cannot conclude that the samples taken at 2 m contained more particles than those obtained at the surface. Shallow coastal waters in Kuwait are usually well mixed, and tides and wind frequently resuspend material within the water column.

Independent validation of EFM counts by electron microscopy, flow cytometry, or other complementary approaches was not available for the historical samples and should be considered in future monitoring. Automated image analysis, such as EpiVirQuant, may further improve particle separation and provide particle-size information [55]. The two-month sampling interval may also have missed short-term changes associated with blooms, tides, or sediment resuspension. In addition, some comparisons had few observations, and missing nutrient measurements reduced the available dataset. These limitations restrict the temporal and ecological resolution of the study. Total VLP counts cannot identify viral hosts or resolve changes in specific virus–host interactions [56], while the Random Forest and GAM analyses identify associations among the measured variables rather than causal relationships [57,58].

## 5. Conclusions

To our knowledge, this study provides the first four-year assessment of virus-like particle (VLP) abundance in Kuwait coastal waters and the northwestern Arabian Gulf. VLP abundance varied among sites, seasons, and water-column positions, showing that viral dynamics cannot be attributed to a single environmental factor. Random Forest and GAM showed that season, nutrients, and dissolved oxygen contributed to the observed variation. The GAM also showed a nonlinear response to dissolved oxygen and lower VLP abundance at depth. These results provide a baseline for marine VLP abundance and environmental variation in Kuwait waters. Future studies combining long-term VLP monitoring with viral metagenomics, microbial community analyses, and direct measurements of viral production and infection will help identify the hosts and processes underlying these patterns.

## Data Availability

The data supporting the findings of this study are available from the corresponding author upon reasonable request.

## Supplementary Materials

The following supporting information can be downloaded at https://www.mdpi.com/article/10.3390/v18090969/s1, Table S1: Results of the envfit analysis of environmental variables fitted to the principal component analysis (PCA); Table S2: Significant Spearman rank correlations between log_10_-transformed virus-like particle (VLP) abundance and environmental variables after Benjamini–Hochberg false discovery rate correction; Table S3: Summary statistics and non-parametric comparisons of log_10_-transformed VLP abundance among sampling sites and water-column positions; Table S4: Dunn’s post hoc pairwise comparisons of log_10_-transformed VLP abundance among sampling sites; Table S5: Spearman rank correlations among nutrient variables included in the Random Forest analysis; Table S6: Comparison of candidate generalized additive models (GAMs); Table S7: Summary statistics for the final GAM; Table S8: Parametric coefficient estimates for the final GAM; Table S9: Summary of smooth-term statistics for the final GAM; Table S10: Concurvity diagnostics for the selected GAM and the sensitivity model excluding Season; Figure S1: Seasonal Spearman correlations between VLP abundance and environmental variables; Figures S2 and S3: Performance and variable importance of the full Random Forest model; Figures S4–S6: Variable importance and performance of the environment-only Random Forest model.

## Abbreviations

VLP	Virus-like particle
DO	Dissolved oxygen
PCA	Principal component analysis
RF	Random forest
GAM	Generalized additive model
ML	Maximum likelihood
REML	Restricted maximum likelihood
AIC	Akaike’s Information Criterion
RMSE	Root mean square error
BH-FDR	Benjamini–Hochberg false discovery rate
