# Supplementary material for: Temporal, Spatial, and Environmental Variation in Virus-like Particle Abundance in the Northwestern Arabian Gulf
Source: Viruses. 2026 Sep 3;18(9):969. doi: 10.3390/v18090969 (PMC13611963; doi:10.3390/v18090969)
Supplement: Supplementary file 1 [file viruses-18-00969-s001.zip › viruses-4511266-supplementary.pdf]

Supplementary Materials

Table S1. Results of the *envfit* analysis of environmental variables fitted to the principal component analysis (PCA).

Environmental variable	PC1	PC2	R^2	Permutation p-value
Dissolved oxygen	0.708	−0.578	0.834	<0.001
Temperature	−0.714	0.559	0.822	<0.001
Salinity	0.499	0.722	0.770	<0.001
Turbidity	−0.650	−0.582	0.761	<0.001
pH	0.119	0.409	0.182	0.002

Table S2. Significant Spearman rank correlations between $\log_{10}$-transformed virus-like particle (VLP) abundance and environmental variables after Benjamini–Hochberg false discovery rate (BH-FDR) correction. Only correlations with adjusted $p < 0.05$ are shown.

Site	Position	Season	Environmental variable	Spearman ρ	Raw *p*-value	Adjusted *p*-value (BH–FDR)	Significance level
BID	Surface	Summer	Turbidity	−0.818	3.39×10^{-5}	0.00152	**
BID	Surface	Summer	Temperature	−0.696	0.00134	0.0299	*
BID	Surface	Winter	NH_4^+	−1.000	<0.0001	<0.0001	***
NUW	Depth	Autumn	Temperature	−0.949	9.58×10^{-5}	0.00321	**
NUW	Depth	Summer	NH_4^+	−1.000	<0.0001	<0.0001	***
NUW	Depth	Summer	Dissolved oxygen	+0.749	0.00132	0.0299	*

Abbreviations: BH–FDR, Benjamini–Hochberg false discovery rate; NH_4^+, ammonium. Significance levels: $p < 0.05$ (*), $p < 0.01$ (**), and $p < 0.001$ (***).

Table S3. Summary statistics and non-parametric comparisons of log_{10}-transformed virus-like particle (VLP) abundance among sampling sites and water-column positions.

Comparison	n	Median log_{10}(VLP)	IQR	Test	Test statistic	df	p-value	Effect size
SUB	90	8.54	0.367					
BID	90	8.43	0.379					
NUW	90	8.63	0.349					
Overall site comparison	270	—	—	Kruskal–Wallis	H = 14.18	2	0.00083	$\varepsilon^2 = 0.046$
Surface	135	8.59	0.451					
Depth	135	8.47	0.383					
Surface vs Depth	270	—	—	Wilcoxon	W = 11173.5	—	0.00132	r = 0.196

Table S4. Dunn's post hoc pairwise comparisons of log_{10}-transformed virus-like particle (VLP) abundance among sampling sites following Bonferroni correction.

Comparison	Z statistic	Raw *p*-value	Adjusted *p*-value
BID vs NUW	−3.73	0.00019	0.00057
BID vs SUB	−1.42	0.155	0.466
NUW vs SUB	2.31	0.0209	0.0627

Raw and Bonferroni-adjusted p-values are reported. Comparisons with adjusted $p < 0.05$ were considered statistically significant.

Table S5. Spearman rank correlations among nutrient variables included in the Random Forest analysis. Spearman's correlation coefficients (ρ), raw *P*-values, and false discovery rate (FDR)-adjusted *P*-values are reported. FDR-adjusted $P < 0.05$ was considered statistically significant.

Variable 1	Variable 2	Spearman ρ	*P*-value	FDR-adjusted P
NH_4^+	NO_2^-	0.494	1.56×10^{-7}	**7.82×10^{-7}**
NH_4^+	NO_3^-	0.192	0.054	0.108
NH_4^+	PO_4^{3-}	0.588	1.01×10^{-10}	**1.01×10^{-9}**
NH_4^+	SiO_3^{2-}	−0.249	0.012	**0.030**
NO_2^-	NO_3^-	0.031	0.762	0.816
NO_2^-	PO_4^{3-}	0.099	0.325	0.464
NO_2^-	SiO_3^{2-}	−0.023	0.816	0.816
NO_3^-	PO_4^{3-}	0.446	2.92×10^{-6}	**9.72×10^{-6}**
NO_3^-	SiO_3^{2-}	0.077	0.442	0.552
PO_4^{3-}	SiO_3^{2-}	−0.146	0.146	0.243

Table S6. Comparison of candidate generalized additive models (GAMs) fitted using maximum likelihood (ML). Models are ranked according to Akaike's Information Criterion (AIC), with ΔAIC < 2 indicating substantial support for competing models.

Model	Model structure	EDF	Adjusted R^2	Deviance explained (%)	Log-likelihood	AIC	ΔAIC	Akaike weight	Rank
**M3**	Environmental variables + Site + Water-column position + Season	**13.29**	**0.200**	**24.89**	**−59.60**	**148.61**	**0.00**	**0.507**	**1**
M5	Environmental variables + Site + Water-column position + Season + Year	14.00	0.194	24.57	−60.02	150.05	1.44	0.246	2
M6	Environmental variables + Site + Water-column position + Cyclic month	12.88	0.196	24.30	−60.40	150.27	1.67	0.220	3
M7	Environmental variables + Site + Water-column position + Cyclic month + Year	12.77	0.176	22.42	−62.89	154.51	5.91	0.026	4
M2	Environmental variables + Site + Water-column position	10.12	0.136	17.52	−69.14	161.39	12.78	0.00085	5
M4	Environmental variables + Site + Water-column position + Year	11.00	0.127	16.95	−69.84	163.68	15.07	0.00027	6
M1	Environmental variables only	6.54	0.009	3.59	−85.06	185.87	37.27	4.10×10^{-9}	7

EDF, estimated degrees of freedom; AIC, Akaike's Information Criterion; ΔAIC, difference in AIC relative to the best-supported model.

Table S7. Summary statistics for the final generalized additive model (GAM) fitted using restricted maximum likelihood (REML).

Metric	Value
Selected model	M3 (Environmental variables + Site + Water-column position + Season)
Distribution	Gaussian
Link function	Identity
Estimation method	REML
Sample size (*n*)	204
Adjusted R^2	0.201
Deviance explained (%)	25.03
RMSE	0.324
Final AIC	148.44

Table S8. Parametric coefficient estimates for the final generalized additive model (GAM) fitted using restricted maximum likelihood (REML). SUB and Surface were used as the reference categories for Site and Water-column position, respectively. Significant effects ($P < 0.05$) are shown in bold.

Predictor	Estimate	SE	*t*	*P*
Intercept	8.550	0.093	92.32	<0.001
Site (BID)	−0.103	0.127	−0.81	0.418
Site (NUW)	0.131	0.135	0.97	0.334
**Sampling Position (Depth)**	**−0.201**	**0.047**	**−4.26**	**<0.001**

Table S9. Summary of smooth-term statistics for the final generalized additive model (GAM).

Smooth term	EDF	Ref. DF	F	*P*
Temperature	1.000	1.000	1.414	0.236
Salinity	1.000	1.000	0.037	0.848
Turbidity	1.000	1.000	0.640	0.425
pH	1.000	1.000	0.974	0.325
Dissolved oxygen	2.463	2.825	3.184	**0.017**

Table S10. Concurvity diagnostics for the selected generalized additive model (GAM) and the sensitivity model excluding Season.

Model	Smooth term	Worst	Observed	Estimate
Selected GAM (with Season)	Temperature	0.997	0.980	0.981
	Salinity	0.960	0.838	0.836
	Turbidity	0.924	0.603	0.594
	pH	0.834	0.596	0.579
	DO	0.960	0.835	0.854
Sensitivity GAM (without Season)	Temperature	0.960	0.919	0.932
	Salinity	0.953	0.835	0.824
	Turbidity	0.904	0.287	0.548
	pH	0.787	0.493	0.483
	DO	0.958	0.614	0.820

Note: Concurvity was assessed using the concurvity() function in the *mgcv* package. Worst, observed, and estimate are the concurvity measures returned by the analysis, with values closer to 1 indicating greater dependence between a model term and the remaining terms. The sensitivity model excluded Season while retaining the same environmental smooth terms, site, and water-column position.

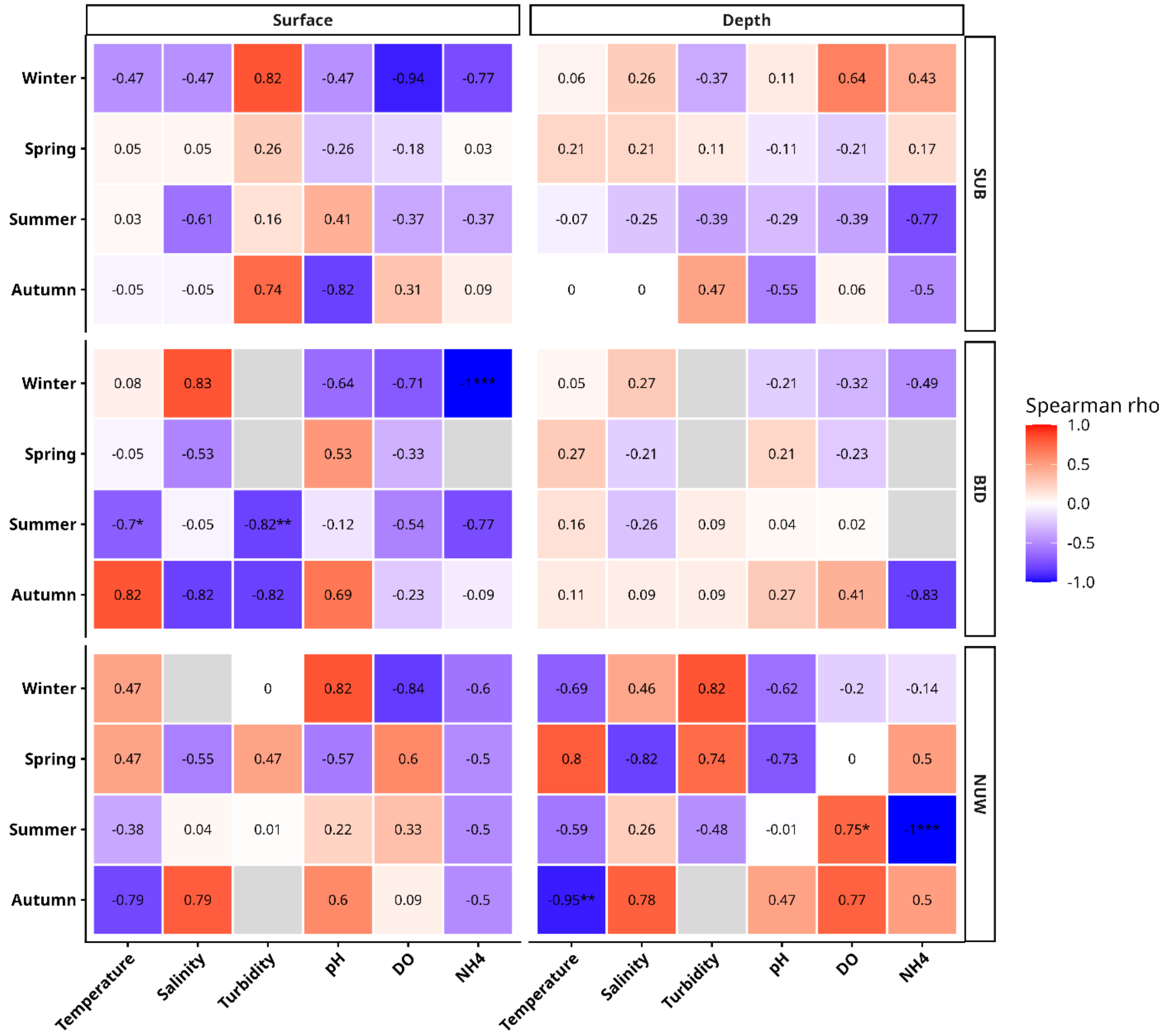

Figure S1. Seasonal Spearman correlation coefficients between virus-like particle (VLP) abundance and environmental variables at surface and depth across the three sampling sites. Correlation coefficients (ρ) are shown within each cell. Asterisks indicate significance after Benjamini–Hochberg false discovery rate correction ($p < 0.05$, **p** < 0.01, and ***p*** < 0.001, based on adjusted p-values).

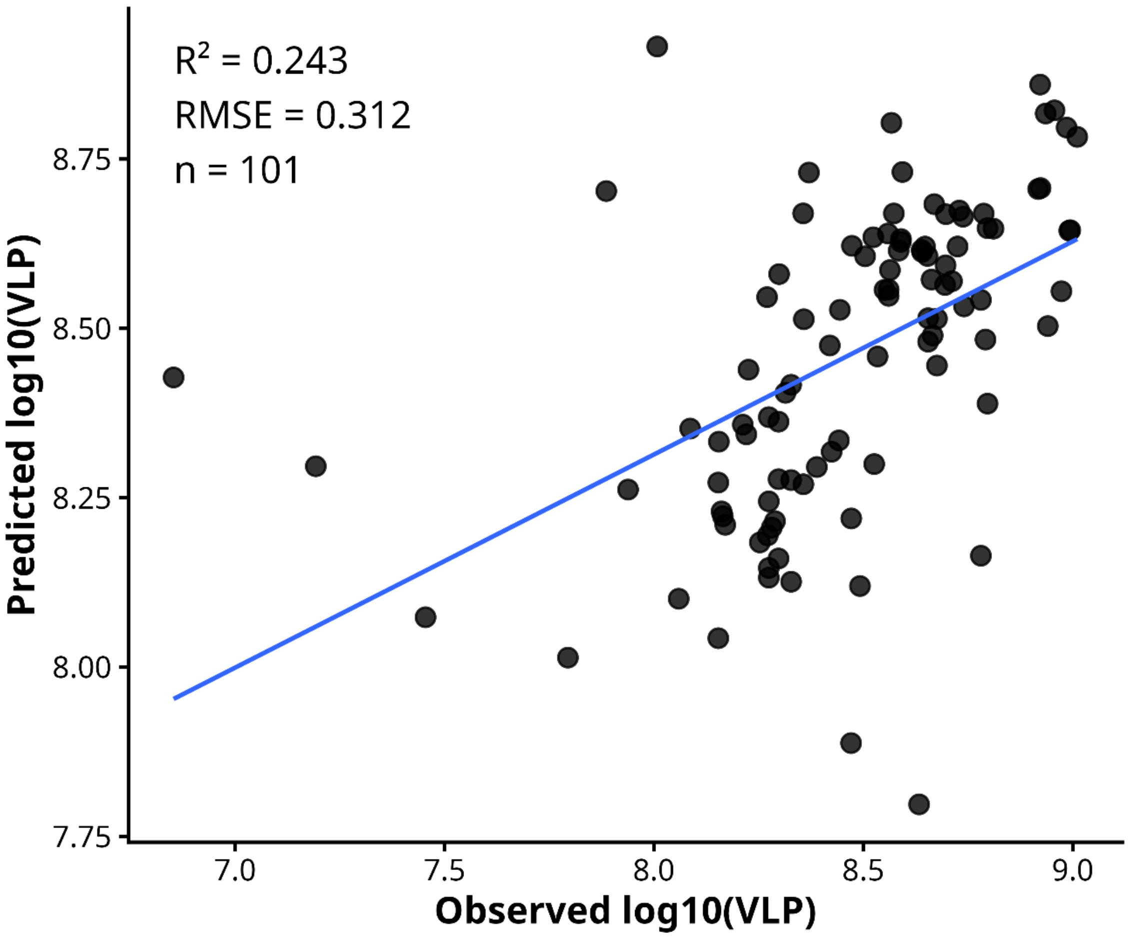

Figure S2. Observed versus predicted log_{10}-transformed virus-like particle (VLP) abundance for the full Random Forest model.

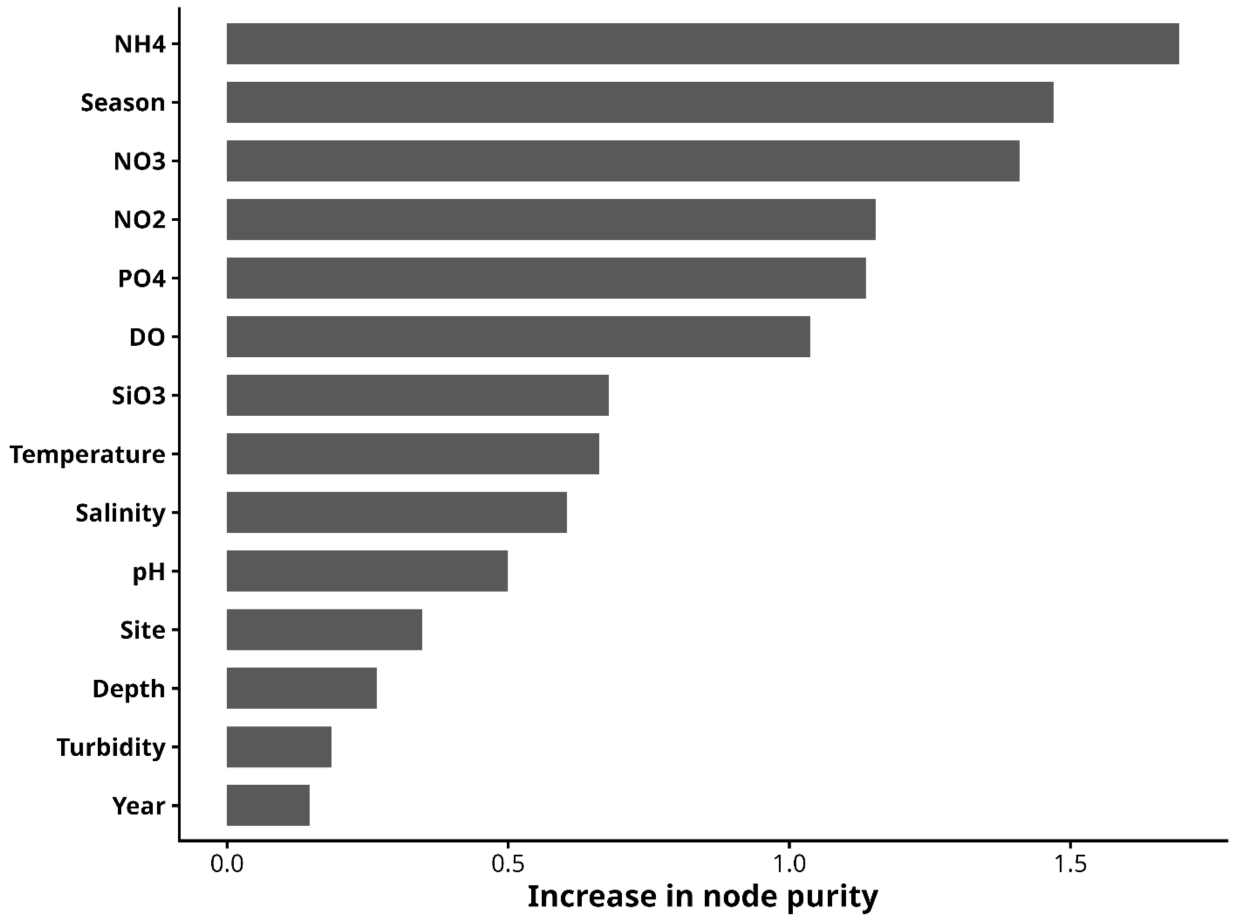

Figure S3. Random Forest variable importance based on increase in node purity for the full model predicting log_{10}-transformed virus-like particle (VLP) abundance. Higher node-purity values indicate greater predictor importance.

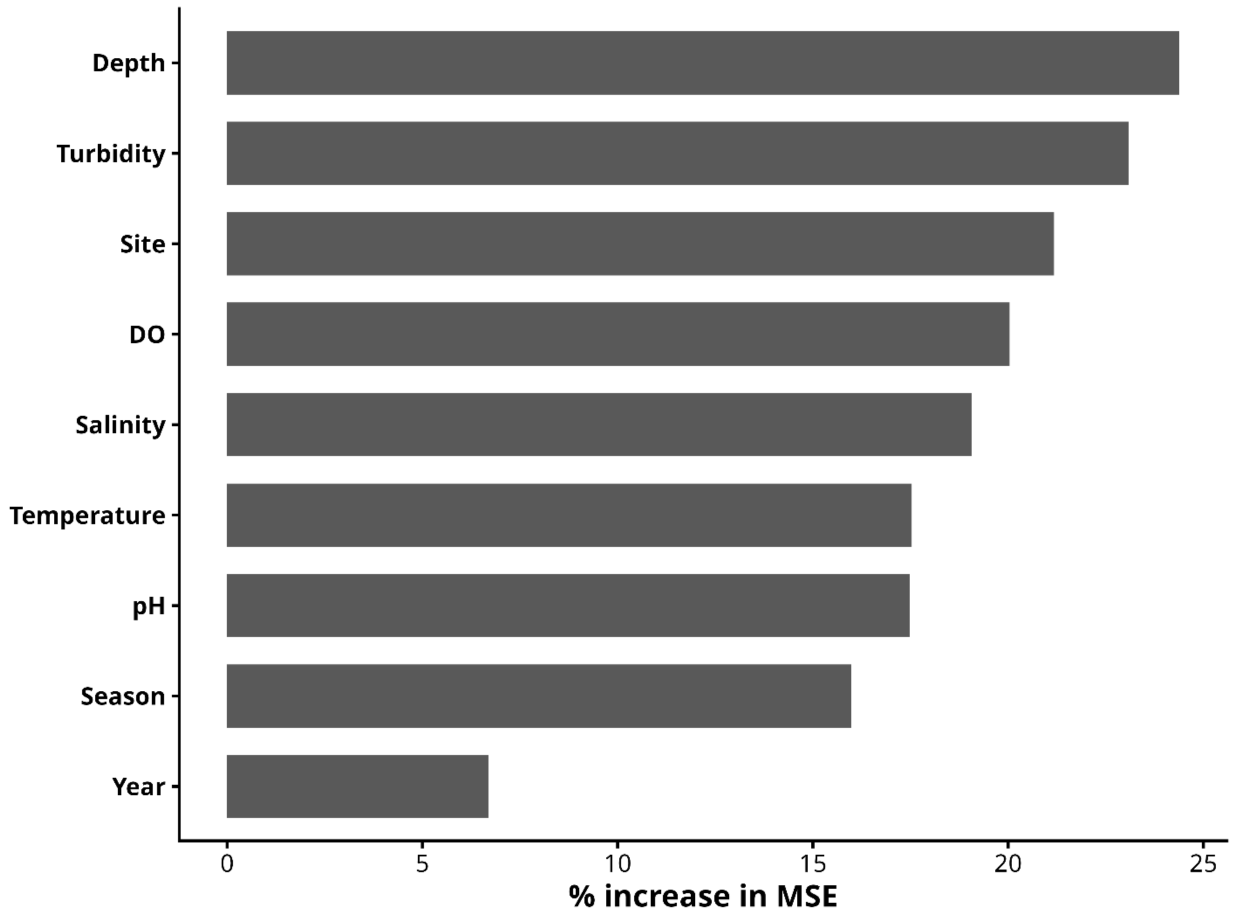

Figure S4. Random Forest permutation variable importance (% increase in mean squared error; %IncMSE) for the environment-only model predicting log_{10}-transformed virus-like particle (VLP) abundance.

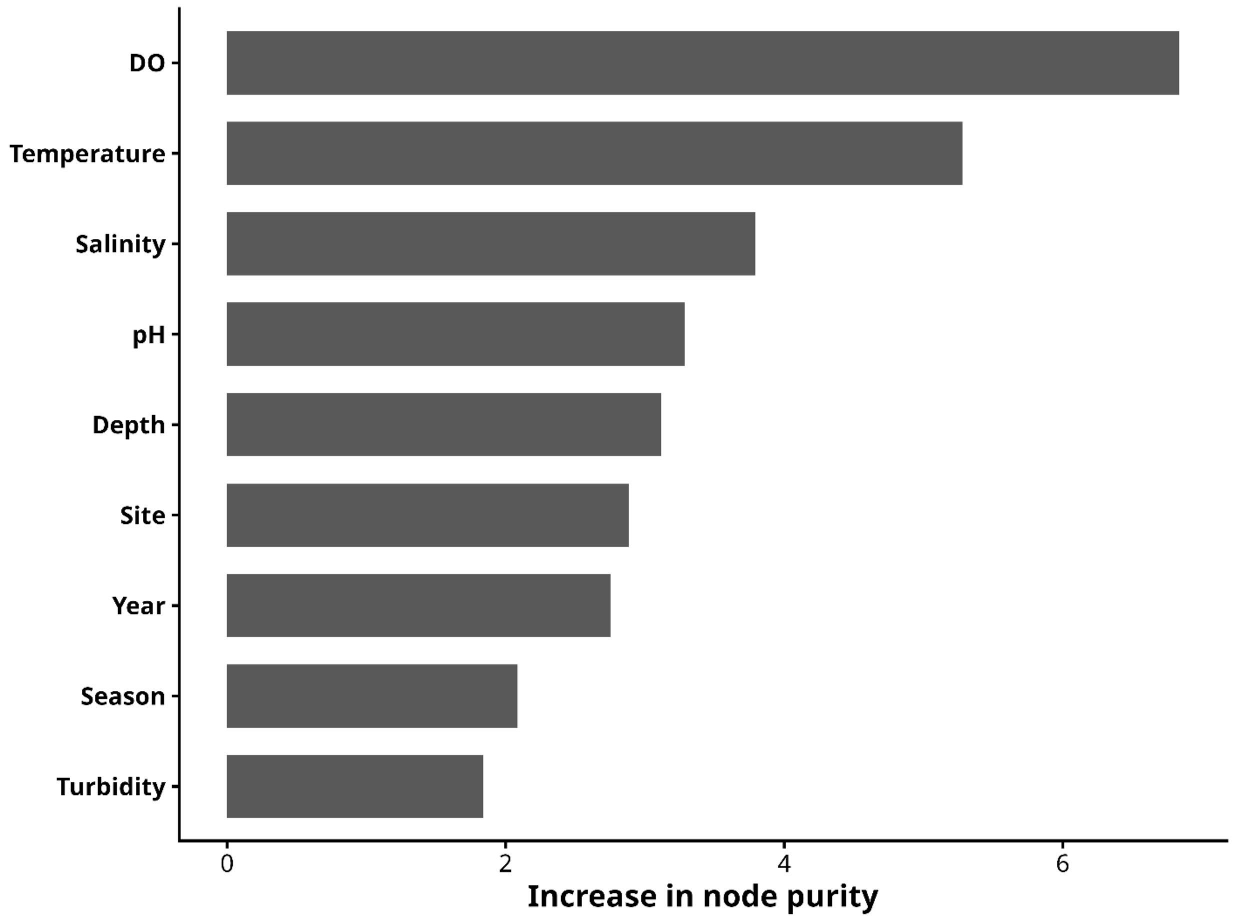

Figure S5. Random Forest variable importance based on increase in node purity for the environment-only model predicting log_{10}-transformed virus-like particle (VLP) abundance. Higher node-purity values indicate greater predictor importance.

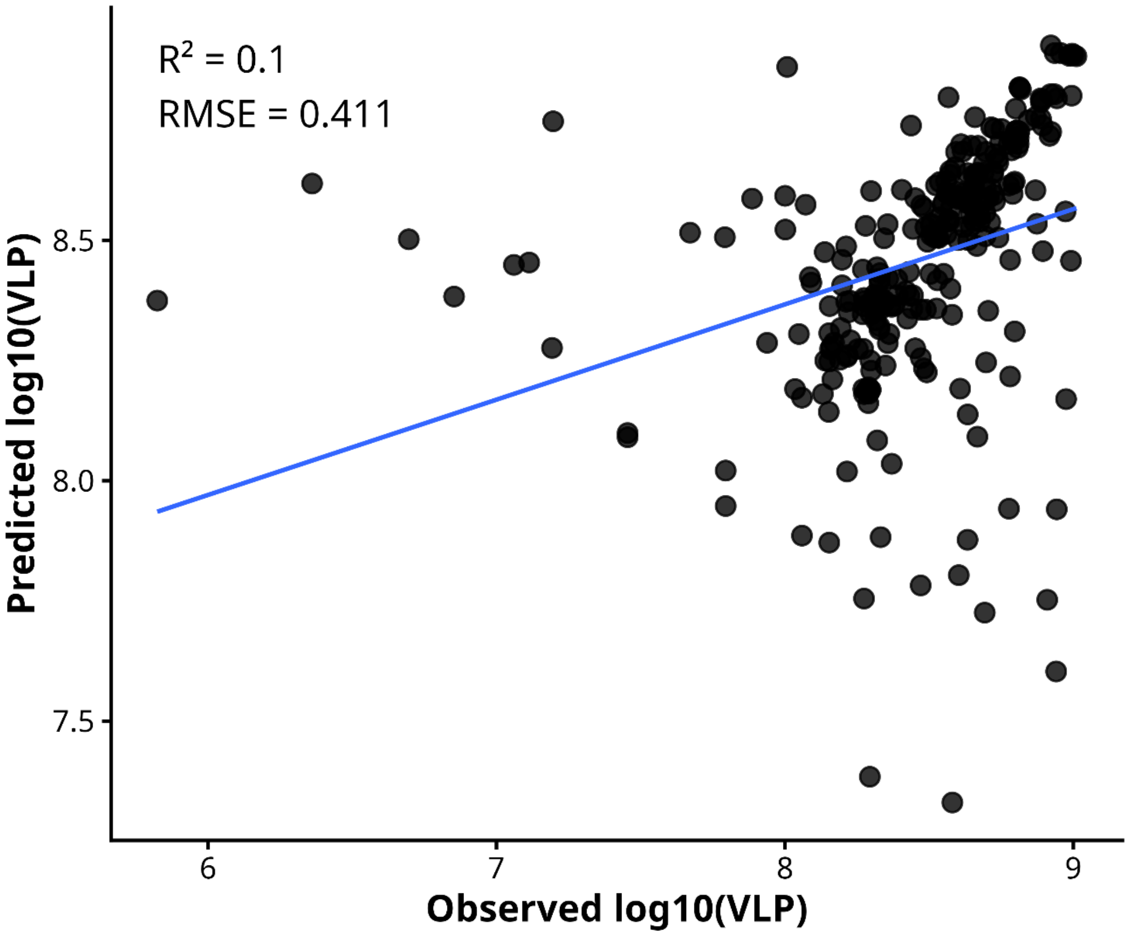

Figure S6. Observed versus predicted log_{10}-transformed virus-like particle (VLP) abundance for the environment-only Random Forest model.
